# Effect of Mesenchymal Stem Cells and a Novel Curcumin Derivative on Notch1 Signaling in Hepatoma Cell Line

**DOI:** 10.1155/2013/129629

**Published:** 2013-08-19

**Authors:** Mohamed Talaat Abdel Aziz, Hussien Mostafa Khaled, Ali El Hindawi, Nagwa Kamal Roshdy, Laila A. Rashed, Dina Sabry, Amira A. Hassouna, Fatma Taha, Walaa Ibrahim Ali

**Affiliations:** ^1^Unit of Biochemistry and Molecular Biology (UBMB), Department of Medical Biochemistry, Faculty of Medicine, Cairo University, Cairo 11956, Egypt; ^2^Department of Medical Oncology, National Cancer Institute, Cairo University, Cairo 11956, Egypt; ^3^Department of Pathology, Faculty of Medicine, Cairo University, Cairo 11956, Egypt

## Abstract

This study was conducted to evaluate the effect of mesenchymal stem cells (MSCs) and a novel curcumin derivative (NCD) on HepG2 cells (hepatoma cell line) and to investigate their effect on Notch1 signaling pathway target genes. HepG2 cells were divided into HepG2 control group, HepG2 cells treated with MSC conditioned medium (MSCs CM), HepG2 cells treated with a NCD, HepG2 cells treated with MSCs CM and NCD, and HepG2 cells treated with MSCs CM (CM of MSCs pretreated with a NCD). Expression of Notch1, Hes1, VEGF, and cyclin D1 was assessed by real-time, reverse transcription-polymerase chain reaction (RT-PCR) in HepG2 cells. In addition, HepG2 proliferation assay was performed in all groups. Notch1 and its target genes (Hes1 and cyclin D1) were downregulated in all treated groups with more suppressive effect in the groups treated with both MSCs and NCD. Also, treated HepG2 cells showed significant decrease in cell proliferation rate. These data suggest that modulation of Notch1 signaling pathway by MSCs and/or NCD can be considered as a therapeutic target in HCC.

## 1. Introduction

Hepatocellular carcinoma (HCC) is the sixth most common malignancy and the third leading cause of cancer deaths worldwide [1]. According to the report of the population-based cancer registry of Gharbiah, the incidence of liver cancer is ranked as the second highest in men and the seventh in women during 2000–2002 [2]. In Gharbiah population-based cancer registry, liver cancer represents 12.7% of male cancers and 3.4% of female cancers [3].

Hepatocellular carcinoma (HCC) is the dominant form of primary liver cancer and is histologically and etiologically distinct from other forms of primary liver cancer [4]. Other types of liver cancer include cholangiocarcinoma, angiosarcoma (or haemangiosarcoma), and hepatoblastoma.

Hepatocellular carcinoma (HCC) is a complex and heterogeneous tumor with multiple genetic aberrations. Several molecular pathways involved in the regulation of proliferation and cell death are implicated in the hepatocarcinogenesis [5].

The Notch1 signalling pathway is a highly conserved developmental pathway, which plays a critical role in cell-fate decision, tissue patterning, and morphogenesis. There is increasing evidence that this pathway is dysregulated in a variety of malignancies and can behave as either an oncogene or a tumor suppressor depending upon cell context [6]. When acting as an oncogene, the Notch1 receptor and signalling pathway are significantly upregulated, which results in increased cellular proliferation, prevention of differentiation, and inhibition of apoptosis [7].

Such a mechanism has been reported in several malignancies including pancreatic cancer, colon cancer, non-small-cell lung cancer, cervical cancer, renal cell carcinoma, and several lymphomas [8]; this signalling pathway therefore represents a potential therapeutic target [9].

Mesenchymal stem cells are known as multipotent and exhibit the potential for differentiation into different cells/tissue lineages [10]. The inhibition of tumor growth by MSCs has been observed in different types of animal models. In experimental models of Lewis lung carcinoma and B16 melanoma (mouse melanoma cell line), Maestroni et al. 1999 [11] first reported that the coinjection of mouse MSCs with tumor cells inhibited primary tumor growth. Although the factors mediating the antitumor activity of MSCs were not identified by the authors, data from that study suggested that they were distinct from inflammatory cytokines. Rat MSCs have the ability to migrate toward glioma cells, to inhibit their proliferation, and, when implanted into the contralateral hemisphere, to migrate to the hemisphere bearing the tumor [12]. When injected directly into the tumor, human skin derived stem cells (hSDSCs) also reduce brain tumor size. hSDSCs were also able to reduce tumor progression in Tyrp1-Tag mice [13].

Curcumin, a phytopolyphenolic pigment derived from turmeric (Curcuma longa), has been shown to have multiple anticancer effects, including inhibition of proliferation, induction of apoptosis, inhibition of angiogenesis, and inhibition of DNA topoisomerase II [14]. Recent studies have demonstrated that Curcumin induces cell death in esophageal cancer cells through modulating Notch signaling [15].

The improvement of the bioavailability of curcumin is a challenge. Bioavailable formulation of curcumin has been developed. A novel water soluble curcumin derivative with conserved natural functional groups (NCD) was developed in our laboratories through covalent modification of the curcumin molecule on sites remote from its natural functional groups.

The present work aimed at evaluating the tumor suppressive effects of MSCs and a novel water soluble curcumin derivative (NCD) on Notch1 signaling in HepG2 cells (hepatoma cell line).

## 2. Methods

### 2.1. Reagents and Chemicals

A novel water soluble curcumin derivative (NCD) was developed through covalent modification of the curcumin molecule on sites remote from its natural functional groups rendering it water soluble. This NCD was presented free of charge to the participating researchers as a personal nonprofit scientific gift to help advancement of cooperation in national medical research, with no rights to use it elsewhere apart from the present study. The novel derivative, (PCT/EG2008/000044, WO 2010/057503, Regional phase European Patent Application no. 08878223) is registered as international patent protected by the rights of “The Patent Cooperation Treaty” and is the personal property of its inventors, Rezq et al., 2008 [16]. 

Histopaque-1077 was purchased from Sigma (St. Louis, MO, USA), Dulbecco's modified Eagle's medium (DMEM) was purchased from Sigma, and fetal bovine serum (FBS; USDA) was purchased from Gibco (Grand Island, NY, USA).

### 2.2. Isolation and Culture of Human Mesenchymal Stem Cells

Under general anesthesia, about 10 mL of bone marrow was drawn from the iliac crest in a syringe containing 1500 U of heparin. Bone marrow was obtained from normal adult donors after informed consent and under a protocol approved by an institutional review board. The isolation of MSCs was performed using the methods of Johnstone et al. [17] and Kadiyala et al. [18]. In brief, the bone marrow aspirate was layered onto Histopaque-1077 (Sigma, St. Louis, MO, USA) and centrifuged at 400 g for 30 min. The collected buffy coat was mixed with 20 mL of Dulbecco's phosphate-buffered saline (DPBS) and centrifuged at 300 g for 5 min. The supernatant was discarded and the cells were washed two more times with DPBS. After determination of cell viability and the number of viable cells by trypan blue staining, the cells were resuspended in Dulbecco's modified Eagle's medium (DMEM) (Sigma) supplemented with 10% fetal bovine serum (FBS; USDA, Gibco, Grand Island, NY, USA) and antibiotics (penicillin 10,000 U/mL, streptomycin 10,000 Lg/mL, amphotericin B 25 *μ*g/mL). The nucleated cells were plated in tissue culture flask at 2.5 × 10^5^/cm^2^ and incubated at 37°C in a humidified atmosphere containing 5% CO_2_. On day 4 of culture, the nonadherent cells were removed along with the change of medium. On day 14, the adherent colonies of cells were trypsinized and counted. Cells were identified as being MSCs by their morphology, adherence, and their power to differentiate into osteocytes [19] and neurocytes [20]. Differentiation into osteocytes was achieved by adding 1–1000 nM dexamethasone, 0.25 mM ascorbic acid, and 1–10 mM beta-glycerophosphate to the medium. Kinetic quantitative determination of alkaline phosphatase (ALP) was carried out in the medium of differentiated cells using a commercial kit provided by Stanbio Laboratory (Boerne, TX, USA). Differentiation into neurocytes was achieved by adding beta-mercaptoethanol, dimethyl sulfoxide, and conditioned medium for neuron induction. Differentiation was confirmed by detection of nerve growth factor (NGF) gene expression in cell homogenate. MSCs from passage 4 were used in this study upon reaching 70%–80% confluence [21].

### 2.3. Cultures of Human Hepatoma (HepG2) Cells

Human hepatoma (HepG2) cells were obtained from ATCC (American Type Culture Collection) and were grown in a sterile 50 cm^2^ tissue culture flask in complete medium containing DMEM supplemented with 10% FBS and antibiotics (100 U/mL penicillin and 100 *μ*g/mL streptomycin) in 95% air/5% CO_2_ at 37°C. Cells were cultured to 100% confluence. Cells from passage 14 were used in this study.

### 2.4. The Cultured HepG2 Cells

The cultured HepG2 cells were divided into 5 groups: 1st HepG2 cells as control cells, 2nd HepG2 cells that were treated with MSCs conditioned medium, and human MSCs that were cultured as described above. The conditioned medium from the MSCs was harvested and clarified by centrifugation. HepG2 cells were treated with a mixture of complete medium containing Dulbecco's modified Eagle's medium (DMEM) supplemented with 10% fetal bovine serum (FBS) and antibiotics (100 U/mL penicillin and 100 *μ*g/mL streptomycin) and MSCs conditioned medium (1 : 1) for 48–96 hours, and the culture medium was replaced every 24 hours.

The third HepG2 cells were treated with NCD (25 *μ*M) and incubated for 24 hours, the fourth HepG2 cells were treated with MSC conditioned medium and NCD (25 *μ*M) and incubated for 24 hours, and the fifth HepG2 cells were treated with MSCs conditioned medium (CM collected from MSCs pretreated with NCD).

First, MSCs were treated with NCD (10 *μ*M) for 24 hours then conditioned medium was collected and was used on HepG2 cells as described above.

HepG2 cells were harvested for assessment of the following: gene expression of Notch1, Hes1, and cyclin D1 genes by quantitative real-time PCR and proliferation rate by MTT cell proliferation assay kit (Trevigen Inc., Gaithersburg, MD, USA).

### 2.5. Real-Time Quantitative Analysis for Notch1, Hes1, and Cyclin D1 Genes Expression in HepG2 Cells

Total RNA was extracted from HepG2 cells using RNeasy purification reagent (Qiagen, Valencia, CA). cDNA was generated from 5 *μ*g of total RNA extracted with 1 *μ*L (20 pmol) antisense primer and 0.8 *μ*L superscript AMV reverse transcriptase for 60 min at 37°C. Real-time qPCR amplification and analysis was performed using Applied Biosystem with software version 3.1 (StepOne, USA). The qPCR assay with the primer sets was optimized at the annealing temperature. All cDNA includes previously prepared samples (for Notch1, Hes1, and cyclin D1 genes expression), with internal control (for *β*-actin gene expression as housekeeping gene).

### 2.6. Primers Sequence of Studied Genes

- *Notch1*:
  – Forward primer: CACTGTGGGCGGGTCC
  – Reverse primer: GTTGTATTGGTTCGGCACCAT
- *Hes1*:
- Forward primer: ATTTTTGGAGTTCTTCACGAAA
- Reverse primer: GAATCCCCCGTCTACCTCTC
- UniSTS: 86110
- *Cyclin  D1*:
  – Forward  primer: GCACAGCTGTAGTGGGGTTCTAGGC
  – Reverse primer: CAGGCGCAAAGGACATGCACACGGC
  – UniSTS: 47064
- *β*-*actin*:
  – Forward primer: GCATTGCTGACAGGATGCAG
  – Reverse primer: CCTGCTTGCTGATCCACATC
  – UniSTS: 272568

### 2.7. Cell Proliferation Assay

Cell proliferation of HepG2 cells in all groups was determined using the MTT (3-[4,5-dimethylthiazol-2-yl]-2.5-diphenyl tetrazolium bromide) cell proliferation kit (Trevigen Inc., Gaithersburg, MD, USA) as per manufacturer's protocol. Briefly, cells were plated in 96-well tissue culture plates in a range of 103–105 cells/well in a final volume of 100 *μ*L of medium and were allowed to attach overnight. The MTT reagent is added (10 *μ*L per well) and the plate is incubated for 2 to 12 h to allow for intracellular reduction of the soluble yellow MTT to the insoluble purple formazan dye. Detergent reagent is added to each well to solubilize the formazan dye prior to measuring the absorbance of each sample in a microplate reader at 550–600 nm. Six wells were used for each group. Cell proliferation was assessed as the percentage of cell proliferation compared to untreated HepG2 as control cells.

## 3. Results

**Figure 1:**
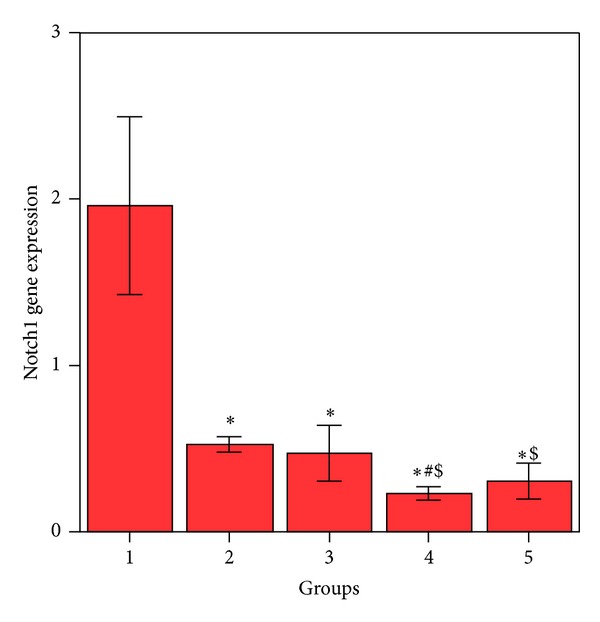
Comparison between the Notch1 gene expressions in HepG2 cell groups. *Values differ significantly from HepG2 control cells. ^$^Values differ significantly from HepG2+MSC CM. ^#^Values differ significantly from HepG2+NCD.

**Figure 2:**
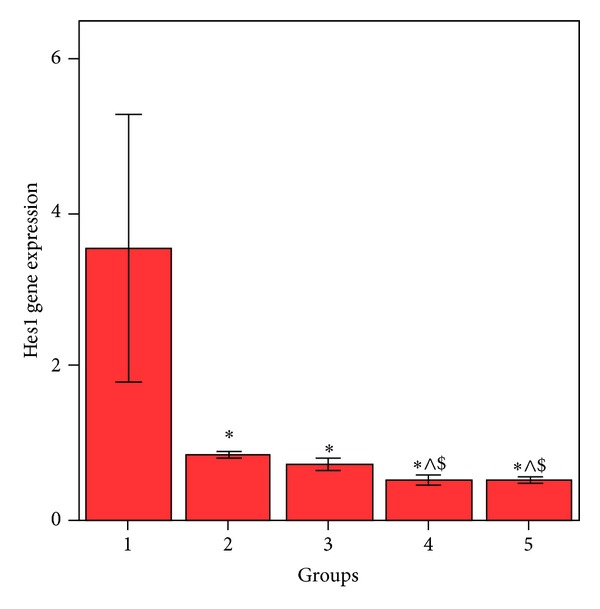
Comparison between the Hes1 gene expressions in HepG2 cell groups. *Values differ significantly from HepG2 control cells. ^∧^Values differ significantly from HepG2+MSC CM.  ^$^Values differ significantly from HepG2+NCD.

**Figure 3:**
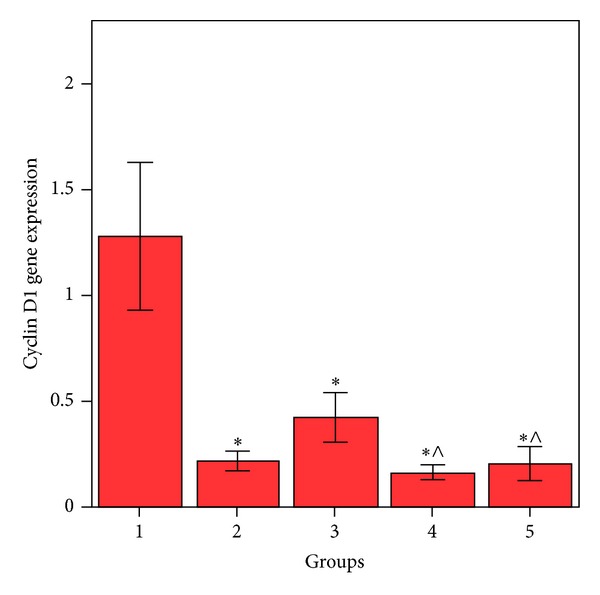
Comparison between the cyclin D1 gene expressions in HepG2 cell groups. *Values differ significantly from HepG2 control cells. ^∧^Values differ significantly from HepG2+NCD.

HepG2 cells treated with MSCs conditioned medium or a NCD showed significant decrease (*P* < 0.05) in Notch1 (Figure 1), Hes1 (Figure 2), and cyclin D1 (Figure 3) gene expression compared to control HepG2 cells.

HepG2 cells treated with both MSCs CM and a NCD showed a significant decrease (*P* < 0.05) in the expression of these genes when compared with the control HepG2 cells; also this group showed significant decrease (*P* < 0.05) in Notch1 and Hes1 gene expression when compared to HepG2 cells treated by MSCs CM and cells treated with a NCD. Cyclin D1 gene expression showed significant decrease (*P* < 0.05) when compared to HepG2 cells treated with a NCD with no significant difference (*P* > 0.05) when compared with HepG2 treated with MSCs CM. HepG2 cells treated with MSCs CM (CM of MSCs pretreated with NCD) showed significant decrease in Notch1, Hes1, and cyclin D1 (*P* < 0.05) when compared to HepG2 control cells. Also Notch1 and Hes1 gene expression was significantly decreased (*P* < 0.05) when compared to the HepG2 cells treated with MSCs CM. Cyclin D1 gene expression was significantly decreased (<0.05) when compared to the HepG2 cells treated with a NCD.

**Figure 4:**
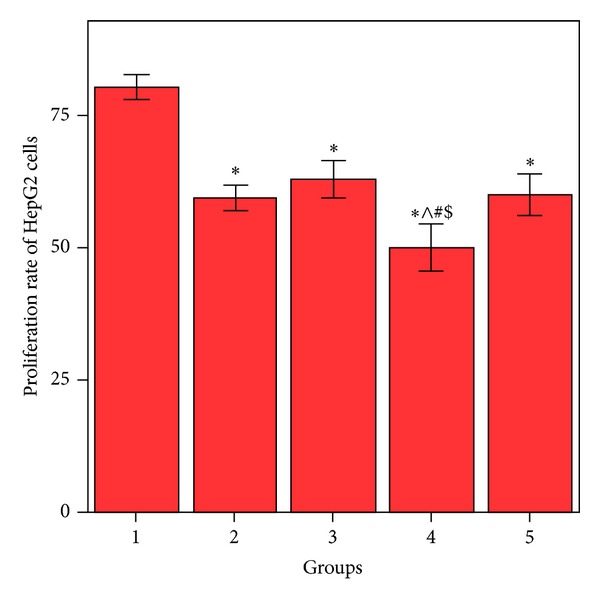
Comparison between MTT proliferation assays of HepG2 cells. *Values differ significantly from HepG2 control cells. ^∧^Values differ significantly from HepG2+MSC CM. ^#^Values differ significantly from HepG2+NCD.  ^$^Values differ significantly from HepG2 cells+MSC CM, (MSCs pretreated with NCD).

MTT proliferation assay of HepG2 cells (Figure 4) showed a significant decrease (*P* < 0.001) in the proliferation rate in the HepG2 cells treated with MSCs CM or NCD compared to the control HepG2 cells with no significant difference between both treated groups (*P* > 0.05). HepG2 cells treated with MSCs CM and NCD showed a significant decrease (*P* < 0.001) when compared to the control HepG2 cells, the HepG2 cells treated with MSCs CM, and the HepG2 cells treated with NCD; it also showed a significant decrease (*P* < 0.001) compared to the HepG2 cells treated MSCs CM (MSCs pretreated with NCD). Also the HepG2 cells treated MSCs CM (MSCs pretreated with NCD) showed a significant decrease (*P* < 0.001) compared to the control HepG2 cells.

## 4. Discussion

 Activation of several signaling pathways has been implicated in human hepatocarcinogenesis [22]. Their relevance resides in their capacity to act as targets for new therapies [23]. Notch signaling plays a critical role in the development and homeostasis of tissues [24] and is frequently deregulated in human malignancies. However, in a limited number of tumor types, including skin and small lung cancer, Notch signaling is antiproliferative rather than oncogenic [25].

Several mechanisms have been suggested to explain the oncogenic role of Notch signaling in solid tumors. Antiapoptotic effects of activated Notch proteins have been linked to the induction of Bcl2 [26] as well as increased signaling through both the phosphatidylinositol 3-kinase (PI3 K) [27] and NF-*κ*B [28] signaling pathways. A positive feedback loop involving Jagged1-induced activation of NF*κ*B signaling [29] and NF-*κ*B-induced transcription of Jagged1 [30] may further enhance the protective effects of notch signaling. Increased cell proliferation in response to notch activation may also promote tumorigenesis. In a kidney epithelial cell line, activated Notch1 promotes cell cycle entry by enhancing CDK2 and cyclin D1 activity, the latter of which may be a direct target of notch [31]. In HeLa cells, Hes1 promotes cell proliferation by repressing transcription of the cyclin-dependent kinase inhibitor p27Kip1 [32]. In 3T3 fibroblasts, notch signaling attenuates p27Kip1 expression by a mechanism independent of transcriptional repression.

Inactivation of notch signaling by novel approaches could be useful for cancer therapy. So, from this aspect, the present study evaluated the effect of MSCs and a novel water soluble curcumin derivative (NCD) on notch signaling in hepatoma cell line (HepG2 cells). Advances in stem cell biology have made the prospect of cell therapy and tissue regeneration a clinical reality [33]. Stem cells and tumor cells share similar signaling pathways that regulate self-renewal and differentiation, including the Wnt, Notch, Shh, and BMP pathways that determine the diverse developmental fates of cells [24]. On the other hand, numerous studies, over the past several years, have evaluated the effects of curcumin and its analogs in several rodent as well as human hepatoma cells [34–38]. 

Treatment of HepG2 cells by MSCs or NCD leads to a significant decrease in the expression of Notch1, Hes1, and cyclin D1 genes which were increased significantly in the malignant cells. Pretreatment of MScs with NCD leads to a more significant decrease in the expression of these genes. This indicates that NCD may improve the function of MSCs when used at low doses.

To evaluate the effect of MSCs soluble factors, MSCs conditioned medium (CM) was used. Application of CM to HepG2 cells decreased gene expression significantly. However, addition of NCD to CM leads to a more significant decrease in the expression of Notch1 and Hes1 but not cyclin D1.

The results of the present work are in accordance with the work of Cantarini et al., [39] who demonstrated Notch-1 and HES1 overexpression in all 15 paired HCC human samples. Furthermore, in a study evaluating 87 resected HCC tumors, Notch1 protein (cytoplasmic) was upregulated in 89% of tumor specimens, when analyzed using immunohistochemical staining and western blot [40]. On the other hand, Qi et al. [41] showed that overexpression of Notch1 induced cell cycle arrest and apoptosis in a single human HCC cell line, SMC7721, but these results were not correlated with human tumor specimens. Ning and coworkers [9] found that high levels of notch intracellular domain (NICD) proteins were present in the human HCC samples compared to the surrounding normal tissues suggesting constitutive activation of the Notch1 and potential oncogenic role of it's pathway in human HCC.

Cyclin D1 is one of the cell cycle regulatory proteins that regulates the G1 to S-phase transition of the cell cycle and functions as a cofactor for several transcription factors in numerous cell lines. This cyclin forms a complex with and functions as regulatory subunit of CDK4 or CDK6, whose activity is required for G1/S transition. Cyclin D1 overexpression has been linked to the development and progression of cancer [42].

As for the mechanisms by which liver regeneration occurs after bone marrow cells transfusion, many mechanisms have been suggested: fusion between hepatocytes and transplanted bone marrow cells has been substantiated as a mechanism by which hepatocytes that carry a bone marrow tag are generated [43], although many studies suggested that cell fusion was not the main mechanism involved in parenchymal repopulation with exogenous cells [44]. Another mechanism may be that the stem cells provide cytokines and growth factors in their microenvironment that promote hepatocyte functions by paracrine mechanisms [43].

Human MSCs home to sites of Kaposi's sarcoma and potently inhibit tumor growth in vivo by downregulating Akt activity in tumor cells that are cultured with hMSCs prior to transplantation in animal tumor models [45]. Furthermore, tumor cells may secrete proteins that can activate signaling pathways that facilitate MSCs migration to the tumor site. Direct transdifferentiation of cells is another mechanism of liver regeneration, although it has not been demonstrated [43]. However, recent observations shed some light on possible mechanisms underlying the observed bone-marrow-derived cells (BMDC) transdifferentiation driven by injured tissues [46].

As regards the effect of MSCs conditioned media, Parekkadan et al. [47] demonstrated that MSC-derived molecules can protect against hepatocyte death and increase survival in Gal-N induced fulminant hepatic failure (FHF). It has been shown that an intravenous bolus of MSC-CM during active disease can reverse organ failure. 

The present work agrees with Qiao et al., [48] who studied the effect of conditioned media from Z3 cells on human H7402 and HepG2 hepatoma cell lines. The researchers found that there were considerably fewer colony-forming units in hepatoma cells treated with conditioned media than those in control cells. These data suggest that conditioned media from Z3 cells may inhibit tumor cell phenotypes via the secretion of soluble factors that are involved in the Wnt signaling pathway.

The present work agrees with Ning and coworkers [9] who reported that downregulation of Notch1 expression by curcumin led to a significant growth inhibition in human HCC cell lines HEP3B, SK-Hep-1, and SNU449.

Subramaniam and coworkers [15] found that curcumin inhibits Notch1 and its ligand Jagged1 in esophageal cancer cells. It also was found that curcumin inhibited the expression of the Notch1 downstream target Hes1. The authors suggested that curcumin treatment inhibits cancer cell proliferation by inhibiting cyclin D1 mRNA and protein expression and increasing expression of p21 protein.

It was observed that levels of cleaved PARP, a well-known apoptotic marker, increased in HCC cells when Notch1 expression was suppressed. Moreover, it was found that the reduction in Notch1 by curcumin or siRNA transfection led to an increase in p21 and a decrease in cyclin D1, a pattern indicative of cell cycle arrest. These results suggest that the downregulation of Notch1 inhibits cell growth through apoptosis and cell cycle arrest in HCC cells [9].

Studies have demonstrated a connection between curcumin, Notch1, and NF-*κ*B. Notch1 signaling pathway has been directly shown to be activated by NF-*κ*B in oral cancer cells [49]. Moreover, curcumin mediated inhibition of Notch1 activation also led to the downregulation of NF-*κ*B and its target genes, including Bcl-2, cyclin D1, vascular endothelial growth factor (VEGF), and matrix metalloproteinase-9 (MMP-9) in oral squamous cell carcinoma cells [14].

Another mechanism by which curcumin downregulates notch signaling is *γ*-secretase inhibition: *γ*-secretase is a multiprotein complex containing an intramembrane cleaving protease. Curcumin treatment resulted in downregulation in the expression of *γ*-secretase complex proteins, Presenilin 1 and 2, Nicastrin, APH1, and PEN2. These data suggest that curcumin mediated downregulation of the notch signaling pathway occurs in part through the inhibition of the c-secretase complex [15].

As for the synergistic effect of MSCs and NCD, Buhrmann et al. [50] found that curcumin treatment may help establish a microenvironment in which the effects of proinflammatory cytokines are antagonized, thus facilitating chondrogenesis of MSC-like progenitor cells in vivo.

 Friedman [51] reported that curcumin in low doses enhanced stem cell proliferation in vitro and proved that curcumin acts synergistically with stem cells in a mouse model of spinal cord injury. Other studies have documented antioxidant and anti-inflammatory effects of micromolar concentrations of curcumin in cultured tumor cell lines as well as normal nonneuronal cells [52].

In the present work, the applications of MSCs CM and NCD to HepG2 cells lead to a significant reduction in the proliferation rate of the cells. The effect was even more significant than either MSCs or NCD.

These results are in accordance with the results of our previous work that studied the effect of human mesenchymal stem cells CM on hepatoma cell line [53]. 

These findings also agree with the study performed by Ning and coworkers [9], who studied the effect of curcumin on the proliferation of human HCC cell lines HEP3B and SK-Hep-1. The researchers reported that curcumin caused clear growth inhibition at day 6 compared to baseline.

## 5. Conclusion

MSCs and/or NCD led to growth inhibition of HepG2 cells due to induction of apoptosis and cell cycle arrest through down regulation of Notch1 signalling. Also, our results suggest that NCD potentiates the antitumor effects of MSCs in HepG2 cells by suppressing Notch1 regulated genes production. These data suggest that modulation of Notch1 signalling pathway by MSCs and/or NCD can be considered as a therapeutic target in HCC. The present study proves also the synergistic effect of NCD and MSCs in the prevention and treatment of HCC.
